# Roles of the α_1B_-Adrenergic Receptor Phosphorylation Domains in Signaling and Internalization

**DOI:** 10.3390/ijms242316963

**Published:** 2023-11-30

**Authors:** David A. Hernández-Espinosa, Rocío Alcántara-Hernández, K. Helivier Solís, J. Adolfo García-Sáinz

**Affiliations:** Departamento de Biología Celular y Desarrollo, Instituto de Fisiología Celular, Universidad Nacional Autónoma de México, Ciudad Universitaria, Ciudad de México 04510, Mexico; fac.ciencias@live.com.mx (D.A.H.-E.); ralcanta@ifc.unam.mx (R.A.-H.); samsonyte09@gmail.com (K.H.S.)

**Keywords:** α_1B_-adrenergic receptor, receptor phosphorylation, receptor desensitization, receptor internalization, phosphorylation sites

## Abstract

The function of the α_1B_-adrenergic receptor phosphorylation sites previously detected by mass spectrometry was evaluated by employing mutants, substituting them with non-phosphorylatable amino acids. Substitution of the intracellular loop 3 (IL3) sites did not alter baseline or stimulated receptor phosphorylation, whereas substitution of phosphorylation sites in the carboxyl terminus (Ctail) or both domains (IL3/Ctail) markedly decreased receptor phosphorylation. Cells expressing the IL3 or Ctail receptor mutants exhibited a noradrenaline-induced calcium-maximal response similar to those expressing the wild-type receptor, and a shift to the left in the concentration–response curve to noradrenaline was also noticed. Cells expressing the IL3/Ctail mutant exhibited higher apparent potency and increased maximal response to noradrenaline than those expressing the wild-type receptor. Phorbol ester-induced desensitization of the calcium response to noradrenaline was reduced in cells expressing the IL3 mutant and abolished in cells in which the Ctail or the IL3/Ctail were modified. In contrast, desensitization in response to preincubation with noradrenaline was unaffected in cells expressing the distinct receptor mutants. Noradrenaline-induced ERK phosphorylation was surprisingly increased in cells expressing IL3-modified receptors but not in those expressing receptors with the Ctail or IL3/Ctail substitutions. Our data indicate that phosphorylation sites in the IL3 and Ctail domains mediate and regulate α_1B_-adrenergic receptor function. Phorbol ester-induced desensitization seems to be closely associated with receptor phosphorylation, whereas noradrenaline-induced desensitization likely involves other elements.

## 1. Introduction

Adrenergic receptors (ARs), a family of G-protein-coupled receptors (GPCRs), mediate the actions of adrenaline and noradrenaline (NA). The α_1_-AR subfamily consists of three members (α_1A_, α_1B_, and α_1D_ subtypes) that are mainly coupled to the G_αq_/phospholipase C pathway to generate inositol (1,4,5)-trisphosphate and diacylglycerol; inositol trisphosphate activates calcium release from intracellular stores, whereas diacylglycerol stimulates protein kinase C [1]. However, the α_1_-AR subtypes have different spatiotemporal mechanisms of internalization and signaling [2]. As expected for a GPCR, the α_1B_-AR comprises seven transmembrane domains joined by three intracellular and three extracellular loops; the amino terminus faces the extracellular space, whereas the carboxyl terminus (Ctail) is in the cytoplasm. 

Receptor phosphorylation is an early event in GPCR action and desensitization, and the specific residues in which the receptors are phosphorylated are critical for receptor localization and signaling, which has been named the “phosphorylation barcode hypothesis”; factors such as the cell type in which the receptors are expressed or the processes that induce phosphorylation are critical determinants of the phosphorylated sites and the functional outcomes [3,4,5].

The pioneering work of Susanna Cotecchia and coworkers employing a mutagenesis/functional response strategy showed that hamster α_1B_-AR phosphorylation sites were localized in a Ctail region (S394, 400, 404, 408, and 410 of the hamster ortholog) [6,7] and that G-protein-coupled receptor kinases and protein kinase C were the main phosphotransferases responsible for phosphorylation [7,8]. Serine/alanine substitutions in the human α_1B_-AR sites (S406, 410, 412A) corresponding to those previously identified as protein kinase C phosphorylation targets in the hamster ortholog decreased desensitization, but receptor phosphorylation was still clearly detected [9], suggesting that additional phosphorylation sites exist and might play functional roles. Using immunopurification/mass spectrometry, we detected phosphorylation sites in human α_1B_-AR under baseline, agonist- and phorbol ester-stimulated conditions. Our data identified a large number of phosphorylation sites located not only in the Ctail (S396, 400, 402, 406, 423, 425, 427, 455, and 470, as well as T387, 392, 420, and 475) but also in intracellular loop 3 (IL3) (S246, 248, 257, 267, and 277, as well as T252, 264, and 268) [10]. We tested the consequences of substituting the phosphorylated residues found in the mass spectrometry studies for non-phosphorylatable amino acids in these domains, i.e., mutants substituting the sites in the IL3, Ctail, or both (IL3/Ctail) (these mutants are depicted in Supplementary Figure S1; for simplicity, mutants are depicted with indications of the domain(s) in which substitutions were made). Our findings provide evidence that phosphorylation sites in the IL3 and Ctail domains play crucial roles in regulating the receptor’s function and localization.

## 2. Results

**Figure 1:**
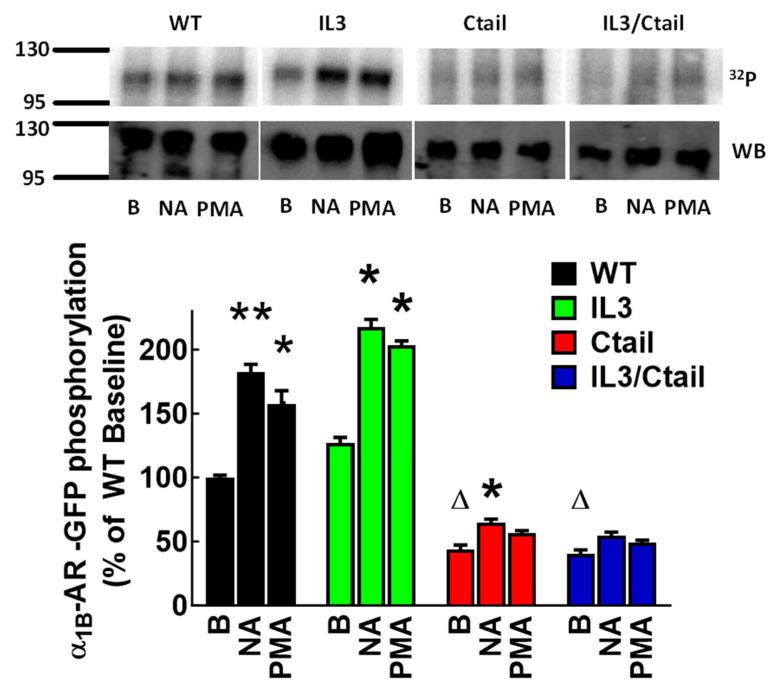
NA- and PMA-induced α_1B_-AR phosphorylation. Cells expressing the WT receptor or the distinct receptor mutants (IL3, Ctail, and IL3/Ctail) were incubated in the absence of any agent (B, baseline) or the presence of either 10 µM NA (2 min) or 1 µM PMA (15 min). Receptor phosphorylation is expressed as the percentage of the WT baseline values. The means are plotted, and error bars indicate the S.E.M. of 3 experiments performed on different days using different cell cultures. ** *p* < 0.01 vs. its respective baseline value; * *p* < 0.05 vs. its respective baseline value; Δ *p* < 0.01 vs. WT baseline value. Representative autoradiographs (^32^P) and Western blots (WB) are presented above the graph.

Cell lines expressing similar levels of the α_1B_-AR constructs (tagged at the Ctail with enhanced green fluorescent protein (eGFP)) as reflected by fluorescence and Western blotting were obtained. The effects of 10 µM noradrenaline (NA) and 1 µM phorbol myristate acetate (PMA) on α_1B_-AR phosphorylation were studied in cells expressing the WT or mutant receptors. The concentration of these agents and the time of stimulation (NA, 2 min; PMA, 15 min) were selected based on preliminary experiments where we observed maximal receptor phosphorylation. As anticipated (Figure 1; Supplementary Figure S2), NA and PMA increased receptor phosphorylation in cells expressing the WT receptor; surprisingly, cells expressing the IL3 mutant showed a similar pattern. The effect of NA was marginally significant, and no significant PMA action was detected in cells expressing the Ctail α_1B_ mutant receptor. No significant change in phosphorylation was induced by these two agents in cells expressing the IL3/Ctail receptor (*p* > 0.9) (Figure 1; Supplementary Figure S2). Baseline α_1B_-AR phosphorylation in cells expressing the Ctail or IL3/Ctail mutants was markedly decreased (but not abolished) compared to that in cells expressing the WT receptor.

**Figure 2:**
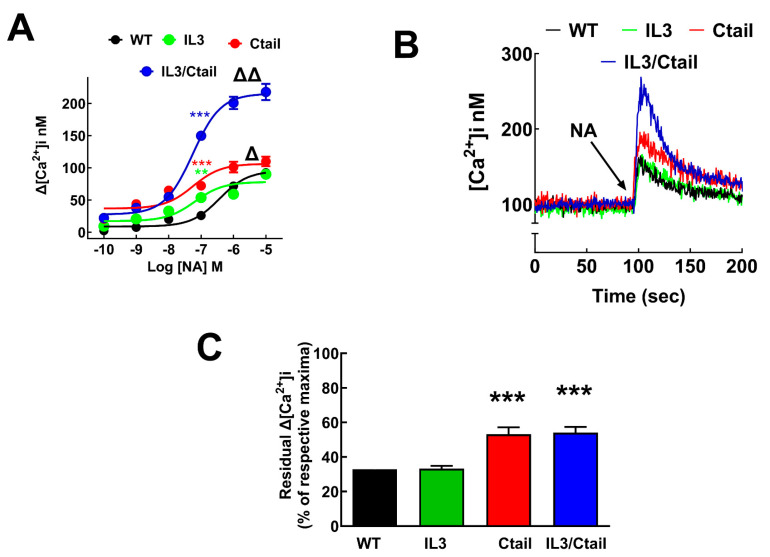
NA-induced calcium response in cells expressing α_1B_-AR mutants. In panel (**A**), cells expressing WT, IL3, Ctail, or IL3/Ctail α_1B_-ARs were incubated with the indicated concentrations of NA, and intracellular calcium was determined. The means are plotted, and error bars indicate the S.E.M. of 10–12 experiments performed on different days using different cell cultures. Where no error bars are shown, they are shown within the symbols. The EC_50_ was smaller (Δ *p* < 0.001, in cells expressing the IL3 or Ctail mutants than in those expressing the WT receptor. Cell expressing the IL3/Ctail mutant showed a bigger calcium response and a marked decrease in EC_50_ compared to the WT (ΔΔ *p* < 0.001 for both parameters). At 100 nM NA, *** *p* < 0.001 (blue, IL3/Ctail; red, Ctail) and ** *p* < 0.005 (green asterisk, IL3) compared to the WT. Panel (**B**) (cells expressing WT, IL3, Ctail, or IL3/Ctail α_1B_-ARs) shows calcium tracings in response to 10 µM NA. In panel (**C**), the residual increases in calcium 100 s after stimulation are indicated. The means are plotted, and error bars indicate the S.E.M. of 10–12 experiments performed on different days using different cell cultures. *** *p* < 0.001 vs. the residual effect observed in cells expressing the WT receptor.

Figure 2A shows that in cells expressing WT α_1B_-ARs, NA increased intracellular calcium in a concentration-dependent fashion; the maximal calcium concentration increase was 90 ± 10 nM, and the EC_50_ was 380 ± 40 nM. The maximal increases in calcium concentration in cells expressing the IL3 and Ctail mutants were similar to those of the wild type, i.e., 67 ± 7 nM (IL3) and 90 ± 8 nM (Ctail). However, a shift to the left in the concentration–response curves was noticed (the EC_50_ values were 65 ± 10 nM (IL3) and 56 ± 7 nM (Ctail); *p* < 0.001 vs. cells expressing the WT receptor) (Figure 2A). Interestingly, cells expressing the IL3/Ctail mutant exhibited a considerably larger maximal response (187 ± 20 nM; *p* < 0.001 vs. the effect of the other receptors at 10 µM NA), as well as a shift to the left in the concentration–response curve when compared to the WT (EC_50_ 60 ± 8 nM; *p* < 0.001 vs. cells expressing the WT receptor). The previous data are the means ± SEMs of 10 concentration–response curves performed with distinct cell cultures in each case.

Analysis of the calcium transients obtained with the different α_1B_-ARs in response to 10 µM NA (Figure 2B) showed that return to baseline after stimulation was slower in cells expressing the Ctail and IL3/Ctail mutants (residual increase 100 s after stimulation, i.e., at ≈200 s, normalized to the maximal responses (100%) of each cell line). In other words, in cells expressing the WT and IL3 constructs, the intracellular calcium concentration decreased to ≈25% of the maximal increase 100 s after stimulation, whereas in cells expressing the Ctail and IL3/Ctail constructs, the decrease at the same time was only to 50% of the maxima (Figure 2C).

**Figure 3:**
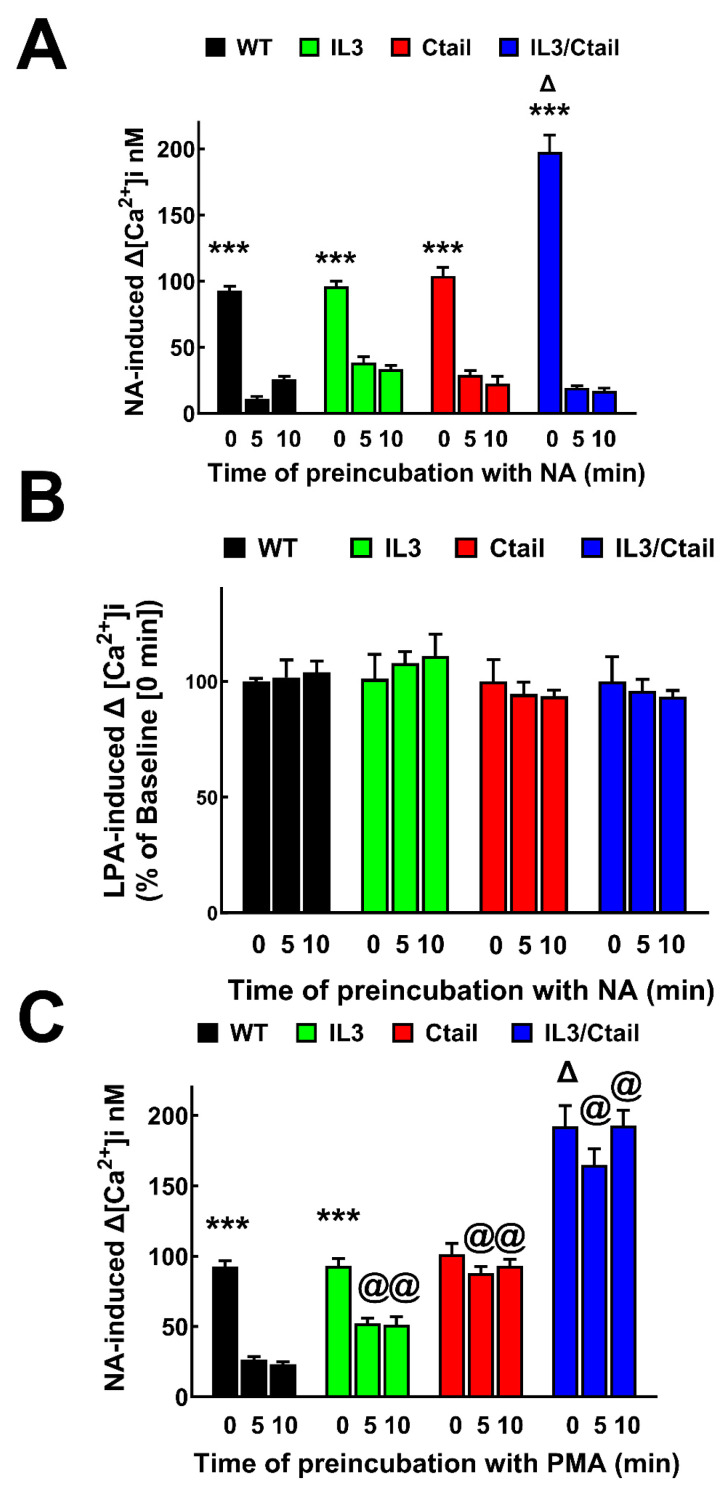
Calcium response to NA in cells preincubated with NA or PMA. In panels (**A**,**B**), cells were preincubated without (time 0) or with 10 µM NA for 5 or 10 min, washed, and subjected to sequential stimulation with 10 µM NA and 1 µM LPA. Panel (**A**) shows the increases in intracellular calcium in response to NA. *** *p* < 0.001 vs. the other columns of the group; Δ *p* < 0.001 vs. no incubation (time: 0 min) of the other receptors. Panel (**B**) shows the increases in intracellular calcium in response to LPA (expressed as the percentage of the baseline value obtained with each cell line). In panel (**C**), cells were preincubated without (time 0) or with 1 µM PMA for 5 or 10 min, then challenged with 10 µM NA. *** *p* < 0.001 vs. the other columns of the group; Δ *p* < 0.001 vs. no incubation (time: 0 min) of the other receptors; @ *p* < 0.001 vs. 5 and 10 min of cells expressing the WT receptor. The means are plotted, and error bars indicate the S.E.M. of 10–12 experiments performed on different days using different cell cultures.

Next, we examined the desensitization of the intracellular calcium response by 10 µM NA (homologous desensitization) or 1 µM PMA (heterologous desensitization) in cells expressing the WT or the different mutants of the α_1B_-ARs (Figure 3). For homologous desensitization, cells were incubated in the absence or presence of NA for 5 or 10 min, then washed three times with buffer to remove the adrenergic amine. Immediately, the cells were resuspended and challenged with 10 µM NA, followed by adding 1 µM lysophosphatidic acid (LPA). LPA is a bioactive lipid capable of increasing intracellular calcium in HEK 293 cells through endogenously expressed LPA receptors and was used as a control. As shown in Figure 3A (representative calcium tracings are presented in Supplementary Figure S3), the data confirmed that cells expressing the IL3/Ctail mutant showed an increased calcium response. Similarly, the return toward baseline of intracellular calcium after NA stimulation of cells expressing the Ctail and IL3/Ctail mutations was slower than that of cells expressing the WT receptor (Supplementary Figure S3). Preincubation with NA essentially abolished (i.e., markedly desensitized) the subsequent response to the adrenergic agent. Interestingly, the response to LPA was not affected at all by pretreatment with NA in cells expressing the WT receptor or any of the mutants (Figure 3B and Supplementary Figure S3). The response to LPA was smaller in cells expressing the Ctail or IL3/Ctail α_1B_-AR mutants.

The effect of preincubation with PMA for 5 and 10 min on the ability of NA to increase intracellular calcium concentration in cells expressing the WT α_1B_-ARs or the mutants differed considerably, as presented in Figure 3C and Supplementary Figure S4. As expected, PMA markedly decreased (≈80–90%) NA action in cells expressing the WT receptor. In cells expressing the IL3 mutant, PMA diminished the α_1B_-AR action to a lesser extent (≈50%); when the responses of cell expressing the IL3 mutant were compared to those expressing the WT, the differences were significant (*p* < 0.001). Interestingly, in cells expressing the Ctail and IL3/Ctail mutants, PMA hardly affected the action of NA, i.e., no desensitization was induced by PMA in cells expressing these mutants (Figure 3C and Supplementary Figure S4).

**Figure 4:**
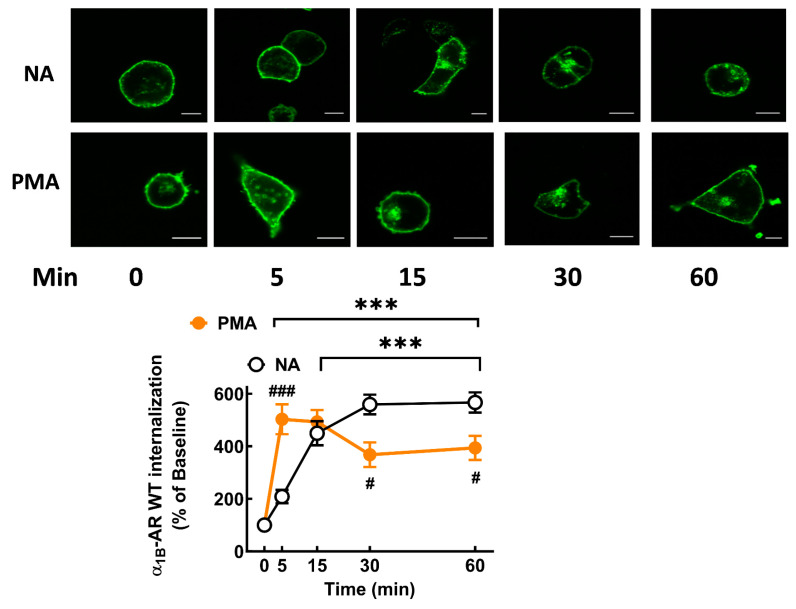
Time course of eGFP-tagged α_1B_-AR internalization in response to NA and PMA in cells expressing the WT receptor. Cells were incubated for the times indicated with 10 µM NA or 1 µM PMA. Internalization is presented as the percentage of baseline intracellular fluorescence. The means are plotted, and error bars indicate the S.E.M. of 30–40 images from 3–4 experiments performed on different days and cell cultures. Representative images are presented above the graph. Bars indicate 10 µm. *** *p* < 0.001 vs. time 0. ^###^ *p* < 0.001 and ^#^ *p* < 0.05 comparing the treatments (PMA vs. NA).

It is well known that α_1B_-ARs internalize in response to agonists and protein kinase C activation [9,11,12,13]. We explored the effects of 10 µM NA and 1 µM PMA on receptor internalization. As shown in Figure 4, both agents induced receptor internalization; PMA-induced internalization was rapid, observed at 2 min, whereas the action of NA reached the maximum at 30 min.

**Figure 5:**
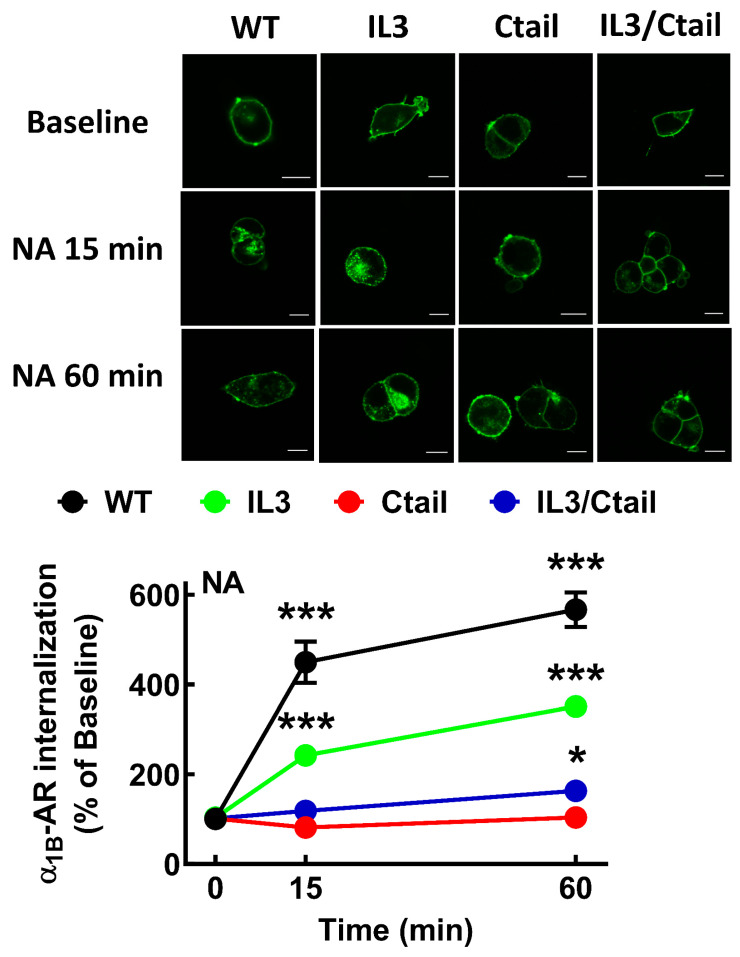
Time course of NA-induced eGFP-tagged α_1B_ internalization in cells expressing WT or mutant α_1B_-ARs. Cells were incubated for 15 or 60 min with 10 µM NA. Internalization is presented as the percentage of baseline intracellular fluorescence. The means are plotted, and error bars indicate the S.E.M. of 25–30 images obtained from 4 experiments performed on different days and cell cultures; where no error bars are presented, they are presented within the symbol. *** *p* < 0.001 vs. time 0; * *p* < 0.05 vs. time 0. Representative images are presented above the graph. Bars indicate 10 µm.

**Figure 6:**
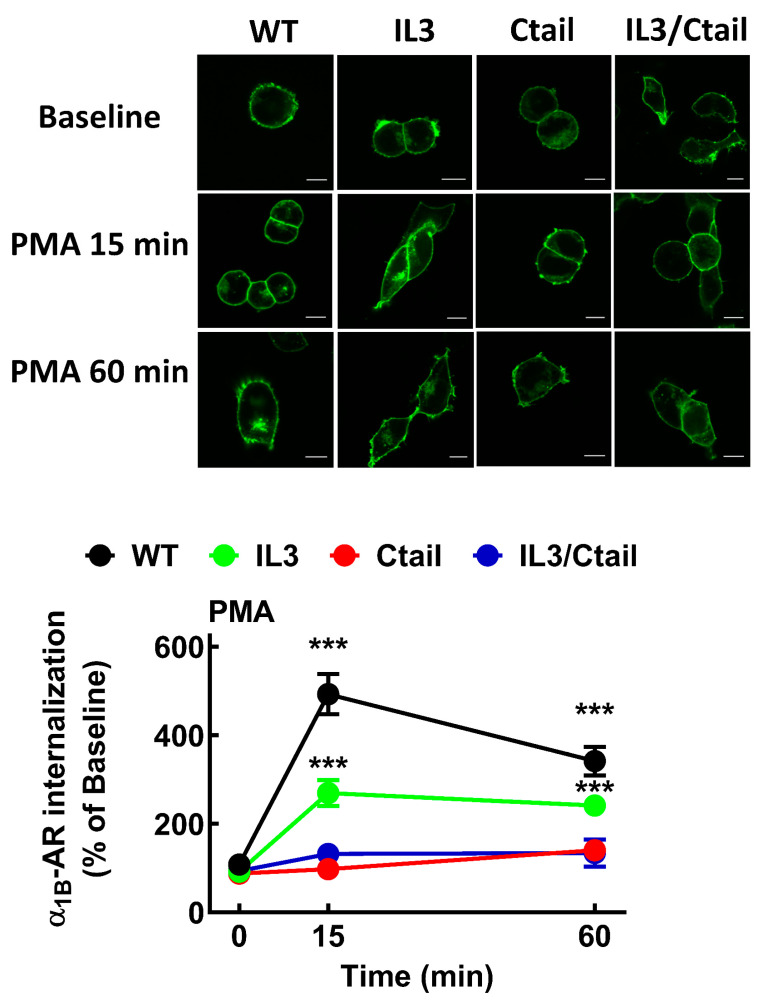
Time course of PMA-mediated α_1B_ internalization in cells expressing WT or mutant eGFP-tagged α_1B_-ARs. Cells were incubated for 15 or 60 min with 1 µM PMA. Internalization is presented as the percentage of baseline intracellular fluorescence. The means are plotted, and error bars indicate the S.E.M. of 25–30 images obtained from 4 experiments performed on different days and cell cultures; where no error bars are presented, they are within the symbol. *** *p* < 0.001 vs. time 0. Above the graph, representative images are presented. Bars indicate 10 µm.

Internalization in response to NA (Figure 5) and PMA (Figure 6) was studied for the different mutants after 15 and 60 min of incubation. All mutations decreased receptor internalization in response to NA and PMA (Figure 5 and Figure 6). IL3 mutant internalization was partially decreased, whereas the Ctail and IL3/Ctail mutants hardly showed any NA- or PMA-induced internalization. NA-induced internalization in WT-expressing cells was more intense at 15 and 60 min as compared to cells expressing the different mutants. Internalization of the IL3 mutant was smaller than that of the WT but significantly bigger than that of the Ctail (*p* < 0.001) and IL3/Ctail (*p* < 0.01) mutants. Similarly, PMA-induced internalization was more pronounced in cells expressing the WT receptor than in cells expressing the IL3 mutant (*p* < 0.05) or the other mutants (*p* < 0.01) at the studied times.

**Figure 7:**
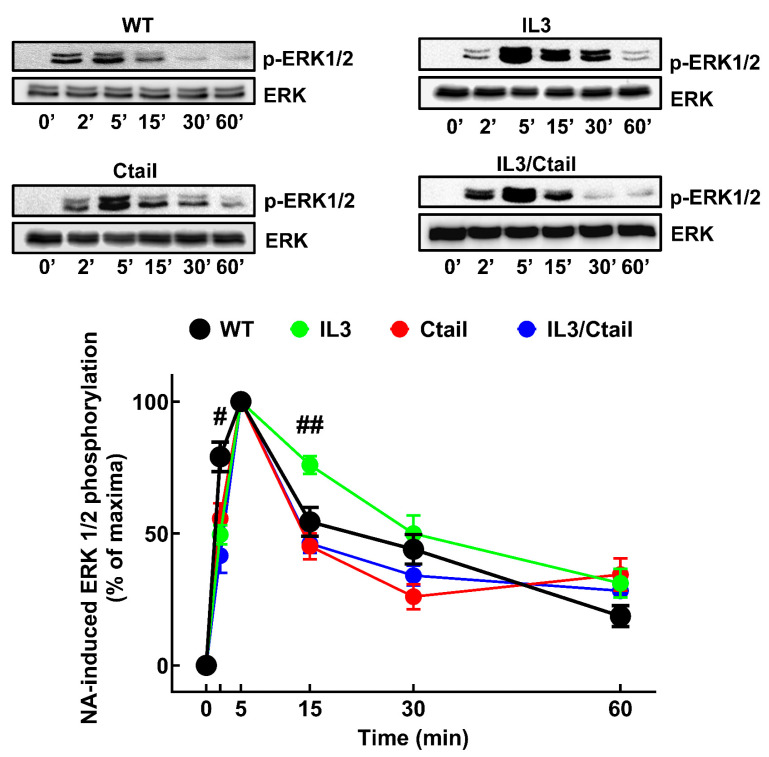
Time course of NA-induced ERK phosphorylation in cells expressing the WT or mutant α_1B_-ARs. Cells were incubated for the times indicated with 10 µM NA. ERK phosphorylation values are the percentage of the maximal effect observed with each cell line. The means are plotted, and error bars indicate the S.E.M. of 9–10 experiments performed on different days and cell cultures; where no error bars are presented, they are presented within the symbol. ^#^ *p* < 0.05 vs. Ctail, *p* < 0.01 vs. IL3, and *p* < 0.001 vs. IL3/Ctail at 2 min; ^##^ *p* < 0.05 vs. WT, Ctail, and IL3/Ctail at 15 min. Representative blots for phospho-ERK (pERK) and total ERK (ERK) are presented above the graph.

**Figure 8:**
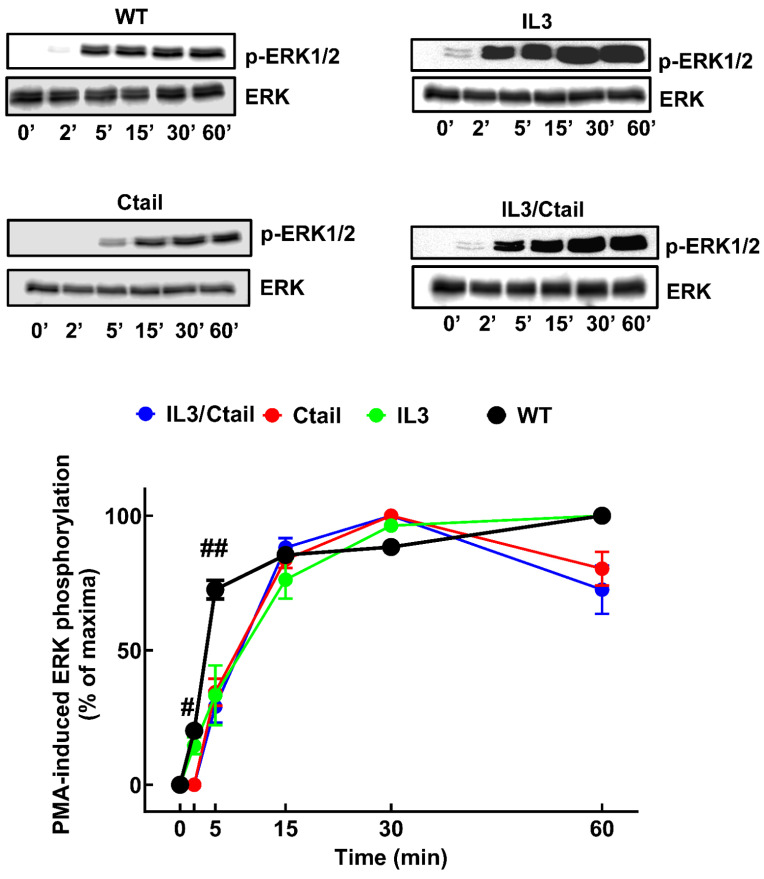
Time course of PMA-induced ERK phosphorylation in cells expressing WT or mutant α_1B_-ARs. Cells were incubated for the times indicated with 1 µM PMA. ^#^ *p* < 0.001 vs. Ctail and IL3/Ctail receptors at 2 min; ^##^ *p* < 0.001 vs. cells expressing the receptor mutants at 5 min. Other indications are the same as in Figure 7.

The effects of NA (Figure 7) and PMA (Figure 8) on ERK 1/2 phosphorylation were studied in cells expressing distinct α_1B_-ARs. No baseline signal was detected, which forced us to present the data as a percentage of the maximal signal obtained for each cell line. Such maxima were observed for all cell lines at 5 min when stimulated by NA, with significant results (*p* < 0.001 vs. baseline) in all cases.

In cells expressing the WT receptor, the phospho-ERK signal decreased progressively, reaching near-baseline values at 60 min. Similar kinetics were observed for the ERK response in cells expressing the other receptor mutants, with only minor changes, as indicated in the figure. When cells were stimulated with PMA (Figure 8), a rapid and significant (*p* < 0.001 vs. baseline) increase in ERK phosphorylation was observed in cell expressing the different α_1B_-ARs, reaching the maxima at 10–15 min and remaining at that level during the experiment (60 min). The kinetics expressing the IL3, Ctail, and IL3/Ctail mutants showed some delay in reaching their maxima compared to cells expressing the WT receptor (Figure 8).

These experiments allowed us to define the individual time courses of ERK phosphorylation of the distinct cell lines but not to define the magnitudes of the responses in comparison with the WT. The relative magnitude of the responses was studied as follows: cell lines expressing the different receptors were incubated (0, 5, and 60 min) in parallel with those of the WT receptor, processed together in the same gels and membranes, and normalized to the WT response at 5 (NA) or 60 min (PMA). Supplementary Figure S5 shows that the effect of NA in cells expressing the IL3 mutant was more prominent than those of cells expressing WT, Ctail, and IL3/Ctail receptors, which were very similar. Supplementary Figure S6 shows that the response to PMA of cells expressing the Ctail receptor was smaller than that of cells expressing the WT receptor. In addition, the data confirmed that the response to PMA was delayed in cells expressing the mutant receptors, evidencing that the response in cells expressing the Ctail receptor was smaller than that of cells expressing the other receptors (Supplementary Figure S6).

A qualitative summary (Table S1) of the functional repercussions of the distinct α_1B_-AR mutations is presented in Supplementary Materials.

## 3. Discussion

In this work, we studied the functional consequences of substituting the amino acids previously found to be phosphorylated by mass spectrometry. The α_1B_-AR phosphorylation studies indicate that complete substitution (IL3/Ctail receptors) essentially eliminates phosphorylation sites and that the treatments did not further affect those remaining. It should be noted that receptor phosphorylation is heterogeneous, i.e., different phosphorylation patterns exist in each cell [14]. The diminished basal, as well as agonist- and PMA-mediated, phosphorylation observed in the Ctail mutant is consistent with findings that the Ctail of α_1B_-AR contains the major phosphorylation sites of this receptor subtype [6,7,15].

The shifts to the left in the concentration–response curves to NA of cells expressing the IL3 or Ctail mutants suggest that phosphorylation sites in both domains negatively modulate the response to the agonist. The shift was more clearly evidenced in cells expressing the IL3/Ctail receptor, and an increased maximal response accompanied it. These data suggest that constraints (i.e., the phosphorylation sites) are removed in this mutant, allowing for full expression of apparent potency and activity. Also consistent with this interpretation were the delays in the return to baseline intracellular calcium levels (residual effects) observed with the IL3/Ctail and Ctail mutants. The IL3 mutant showed only a slight effect on this parameter, which suggests that the leading role likely resides in the Ctail domain. The phosphorylation sites in IL3 play a significant but secondary role, as evidenced when the IL3/Ctail mutant was studied.

The effect of the distinct α_1B_-AR phosphorylation-site substitutions on ERK 1/2 phosphorylation provided some interesting findings. NA-induced ERK phosphorylation was not notably altered in cells expressing the Ctail mutant. There seems to be no clear correlation between agonist-induced internalization and ERK phosphorylation, in agreement with previously published data [2]. Interestingly, cells expressing the IL3 substitutions exhibited an increased response that decreased in parallel with cells expressing the WT receptor. Cells expressing the IL3/Ctail mutant did not share the increased response with those expressing the IL3 mutant; this, again, suggests a complex interaction between these domains to determine the functional response. It has been previously observed that truncation of the Ctail markedly affects NA-induced ERK phosphorylation and receptor internalization [16], and our results show some similarities with those findings. The authors of [16] showed that agonist activation of hamster α_1B_-ARs induced transient ERK phosphorylation dependent on protein kinase C. Receptor Ctail truncation after amino acid 420 allowed for NA-induced ERK phosphorylation, whereas truncation at sites closer to the seventh transmembrane domain blocked the effect. Comparing these data with the mass spectrometry findings [10] and the present data, we suggest that the phosphorylation sites proximal to the seventh transmembrane domain play a significant role in ERK phosphorylation, whereas those more distal might be secondary.

Our interpretation of the effect of the different mutants on PMA-induced desensitization of the α_1B_-AR calcium response to agonist is consistent with the Ctail playing a significant role in heterologous desensitization, strongly suggesting a collaboration with the phosphorylation sites in the IL3 domain.

In contrast, the interpretation of the data of the different mutants on homologous desensitization (agonist-induced desensitization) is much more complex. The results showed marked homologous desensitization in cells expressing any of the studied mutants, including the IL3/Ctail mutants. One possible explanation for these results is that unidentified phosphorylation sites may participate in the effect. We consider this possibility unlikely, but it cannot be discarded. There is evidence of phosphorylation-independent GPCR desensitization (reviewed in [17,18]). Agonist activation might induce receptor sequestration of components of the signaling pathway, such as G proteins, in an inactive state [19,20]. Depletion of calcium from intracellular stores is another possibility. However, the data showing a rise in intracellular calcium in response to the independent agent, LPA, strongly oppose this possibility.

The current approach of substituting amino acids found to be phosphorylated in large domains (such as IL3 or Ctail) for non-phosphorylatable ones has allowed us to propose that for some actions, both domains participate. Such suggestions require experimental definitions of the specific roles of sites or clusters of phosphorylation sites. Mutations can address this aspect, but structural work (crystallography/cryo-electron microscopy) and molecular dynamic analyses are likely to be needed. Our work provides an initial framework according to which such studies could be performed.

## 4. Materials and Methods

### 4.1. Materials

The sources of materials are essentially those described previously [9,10]. (−)-Noradrenaline (NA), phorbol 12-myristate 13-acetate (PMA), 1-oleoyl-sn-glycerol 3-phosphate (LPA), and dl-propranolol were obtained from Sigma-Aldrich Chemical-Merck (Darmstadt, Germany). [^32^P]Pi (8500–9120 Ci/mmol) was obtained from Perkin-Elmer Life Sciences (Shelton, CT, USA) Dulbecco’s modified Eagle medium, fetal bovine serum, trypsin, antibiotics, and Fura-2AM were purchased from Invitrogen-Life Technologies (Carlsbad, CA, USA). Polyethyleneimine was obtained from PolyScience (Niles, IL, USA). Nitrocellulose membranes were obtained from Bio-Rad (Hercules, CA, USA), and SuperSignal West Pico Chemiluminescence kits were obtained from Thermo Fisher Scientific (Austin, TX, USA). Agarose-coupled protein A was obtained from Merck-Millipore, Darmstadt, Germany)). Antiphospho-ERK 1/2 (Thre202/Tyr204) (catalog number 9101S, lot 30) and anti-total ERK (p42/44) antibodies (catalog number 4695, lot 35) were obtained from Cell Signaling Technology (Danvers, MA, USA), and monoclonal anti-GFP was obtained from Clontech-Takara (catalog number 632381, lot A5033481) (Mountain View, CA, USA), with polyclonal anti-GFP generated in our laboratory [9,10]. Secondary antibodies were purchased from Zymed (Thermo Fisher Scientific (Austin, TX, USA)) and Jackson ImmunoResearch (West Grove, PA, USA), including the peroxidase AffiniPure goat anti-mouse IgG light-chain antibody. Antibody dilutions were 1:1000 for primary antibodies and 1:10,000 for secondary antibodies. HEK293 cells were obtained from the American Type Culture Collection (Manassas, VA, USA).

### 4.2. Receptor Mutants, Cells, and Stable Transfections

The plasmid for the expression of human α_1B_-AR fused at the Ctail with eGFP was previously described [21]. The wild-type (WT) α_1B_-AR construct was employed as a template to generate the different mutants used in this study. Mutagenex Inc. performed mutagenesis, which was confirmed by sequencing. The following mutants were studied: (a) a mutant in which the residues detected to be phosphorylated at IL3 were changed for non-phosphorylatable amino acids (S246, 248, 257, 267, and 277A, as well as T252, 264, and 268V (IL3)), (b) a mutant in which the residues that were found to be phosphorylated at the Ctail were changed to non-phosphorylatable amino acids (S396, 400, 402, 406, 423, 425, 427, 455, and 470A, as well as T387, 392, 420, and 475V (Ctail)), and (c) a mutant in which the residues found to be phosphorylated at both the IL3 and Ctail were changed to non-phosphorylatable amino acids (IL3/Ctail). The cell lines exhibited similar receptor construct expression, as reflected by eGFP expression and Western blotting against eGFP. HEK293 cells were transfected with these plasmids and subjected to selection with G418. Resistant cells were isolated, and cell lines were selected according to two criteria: good expression of the constructs, as evidenced by eGFP expression, and strong calcium response to NA. Cells were routinely grown in Dulbecco’s modified Eagle medium supplemented with 10% fetal bovine serum, 100 μg/mL streptomycin, 100 U/mL penicillin, 0.25 μg/mL amphotericin B, and 300 ng/mL G418. In all experiments using NA, 1 μM propranolol was also added to avoid β-adrenergic action; the β-blocker did not alter baseline parameters by itself.

### 4.3. Receptor Phosphorylation

Receptor phosphorylation was performed as previously described [9]. In brief, cells cultured in six-well plates were incubated for 3 h in phosphate-free Dulbecco’s modified Eagle medium supplemented with 50 μCi/mL [^32^P]Pi. Labeled cells were stimulated as indicated, washed with ice-cold phosphate-buffered saline, and solubilized for 1 h in lysis buffer as described previously [9]. The extracts were centrifuged, and the supernatants were incubated overnight with protein A-agarose and the anti-eGFP antiserum. Samples were subjected to SDS-PAGE, transferred onto nitrocellulose membranes, and exposed for 18–24 h. The amount of phosphorylated receptor was assessed by PhosphorImager analysis using the ImageQuant program. In all the experiments, cells expressing the WT receptor were included (see Supplementary Figure S2) to properly compare the distinct mutants’ phosphorylation. The average of the WT baseline values was considered to be 100%, and error bars correspond to the dispersion of these values. Western blotting for loading controls was performed using a commercial monoclonal anti-eGFP antibody.

### 4.4. Intracellular Calcium Determinations

Intracellular calcium concentrations were assessed as previously described [9]. Briefly, cells starved of serum for 2 h were loaded with 2.5 μM Fura-2/AM for 1 h at 37 °C and washed to eliminate unincorporated dye. Fluorescence measurements were performed at 340 and 380 nm excitation wavelengths and a 510 nm emission wavelength, with a chopper interval set to 0.5 s, using an Aminco-Bowman Series 2 Luminescence Spectrometer. Intracellular calcium levels were calculated as described by Grynkiewicz et al. [22]. The baseline intracellular free calcium concentration varied between 80 and 120 nM (this variation is likely due to dye leakage from the cells). The curves were normalized to a baseline of 100 nM in all the tracings shown to facilitate comparison.

### 4.5. Receptor Internalization

Cells expressing the α_1B_-AR constructs were incubated for the times indicated, then fixed, and images were obtained. The plasma membrane was delineated utilizing differential interference contrast imaging. Each cell’s intracellular α_1B_-AR-eGFP fluorescence (i.e., excluding the plasma membrane) was quantified as the “integrated density”, employing ImageJ software (https://imagej.net/ij/download.html; accessed on 20 July 2022). The procedure is described in detail in “Corrected total cell fluorescence” (in [23] and in The Open Lab Book (https://theolb.readthedocs.io/en/latest/imaging/measuring-cell-fluorescence-using-imagej.html; accessed on 20 July 2022)). Usually, 8–10 images of the indicated number of different cultures were taken for each condition. Bars in all microscopic images indicate 10 µm.

### 4.6. ERK 1/2 Phosphorylation

Cells were serum-starved for 4 h. After stimulation with the indicated agents, cells were washed with ice-cold phosphate-buffered saline and lysed with the buffer used in the phosphorylation experiments. Lysates were centrifuged, and SDS-PAGE separated proteins in supernatants. Proteins were electrotransferred onto nitrocellulose membranes and immunoblotted to determine total and phospho-ERK 1/2 levels, using the same membranes. Membranes were subjected to a first Western blotting procedure using the anti-pERK 1/2 antibody and analyzed. The next day, the membranes were subjected to protein stripping using a buffer containing 0.1% Tween 20 and 100 mM glycine (pH 2.5) for 2 h, followed by two washes with PBS (pH 7.4), and subjected to a second Western blotting using the anti-total ERK 1/2 antibody. No baseline phospho-ERK was detected; therefore, the maximal response for each α_1B_-AR construct-expressing cell line was considered to be 100% for data normalization. Additionally, samples from cells expressing different receptor constructs were run in parallel in the same gel and blotted together to adequately compare differences between WT and mutant responses. In this case, maximal ERK phosphorylation in cells expressing the WT receptor was considered to be 100% (these data are presented in the Supplementary Material, and normalization is indicated on the abscissa). Western blots were performed by exposing the membranes to X-ray films (three distinct times) and analyzed with a C-DiGit (Model 3600) and a LI-COR scanner, obtaining images at different exposure times, in addition to densitometric quantification.

### 4.7. Statistical Analysis

Data are presented as the mean ± standard error of the mean. Statistical analysis of comparable groups was performed using an ordinary one-way ANOVA with the Bonferroni post test. GraphPad Prism software (version 8.4.3) was employed for these analyses. A value of *p* < 0.05 was considered statistically significant.

## Data Availability

The data presented in this study are available upon request from the corresponding author.

## Supplementary Materials

The following supporting information can be downloaded at: https://www.mdpi.com/article/10.3390/ijms242316963/s1.
