# Supplementary material for: Roles of the α1B-Adrenergic Receptor Phosphorylation Domains in Signaling and Internalization
Source: Int J Mol Sci. 2023 Nov 30;24(23):16963. doi: 10.3390/ijms242316963 (PMC10707169; doi:10.3390/ijms242316963)
Supplement: Supplementary file 1 [file ijms-24-16963-s001.zip › ijms-2674747-supplementary.pdf]

SUPPLEMENTARY MATERIAL

Figure S1. Cartoon showing the substitutions performed in the α_{1B}-AR.

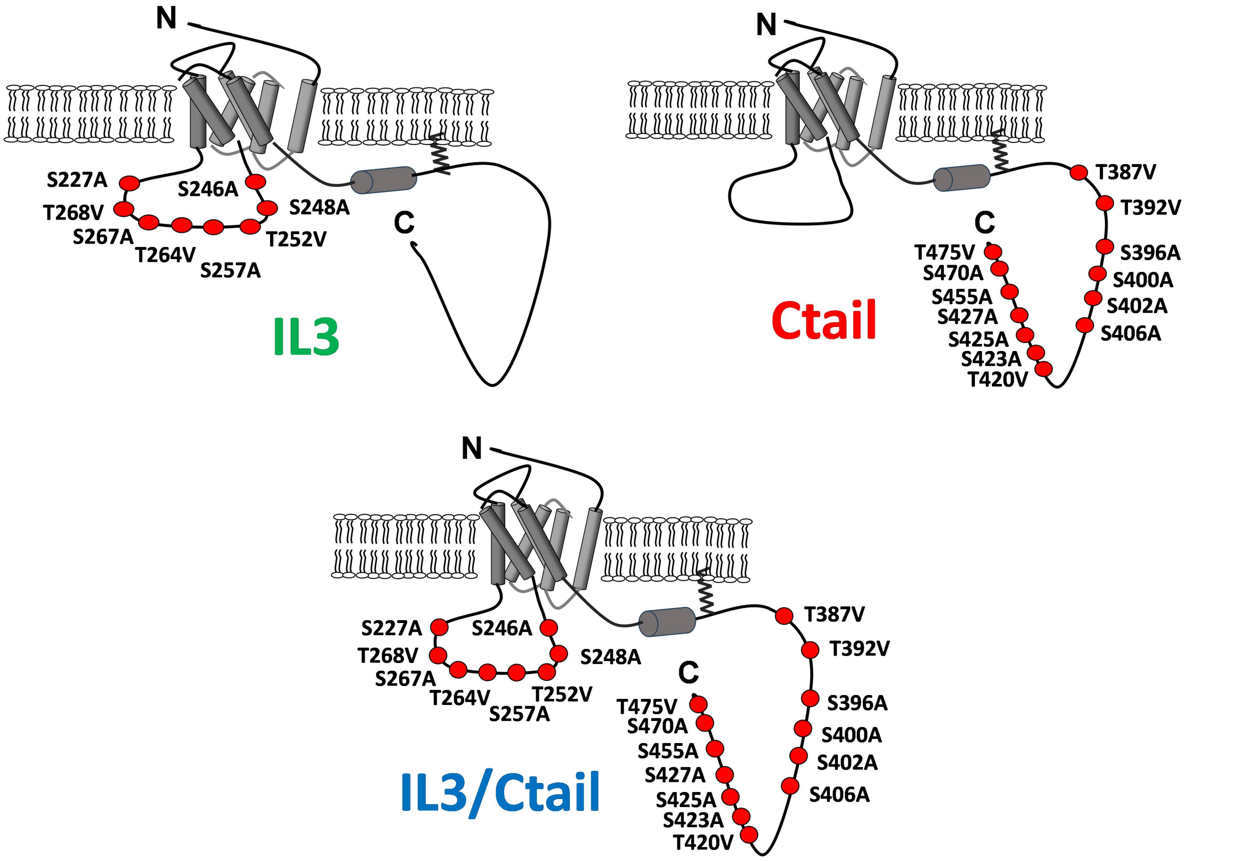

Figure S2 Representative autoradiograph of the phosphorylation of the WT and mutant α_{1B}-ARs run in parallel.

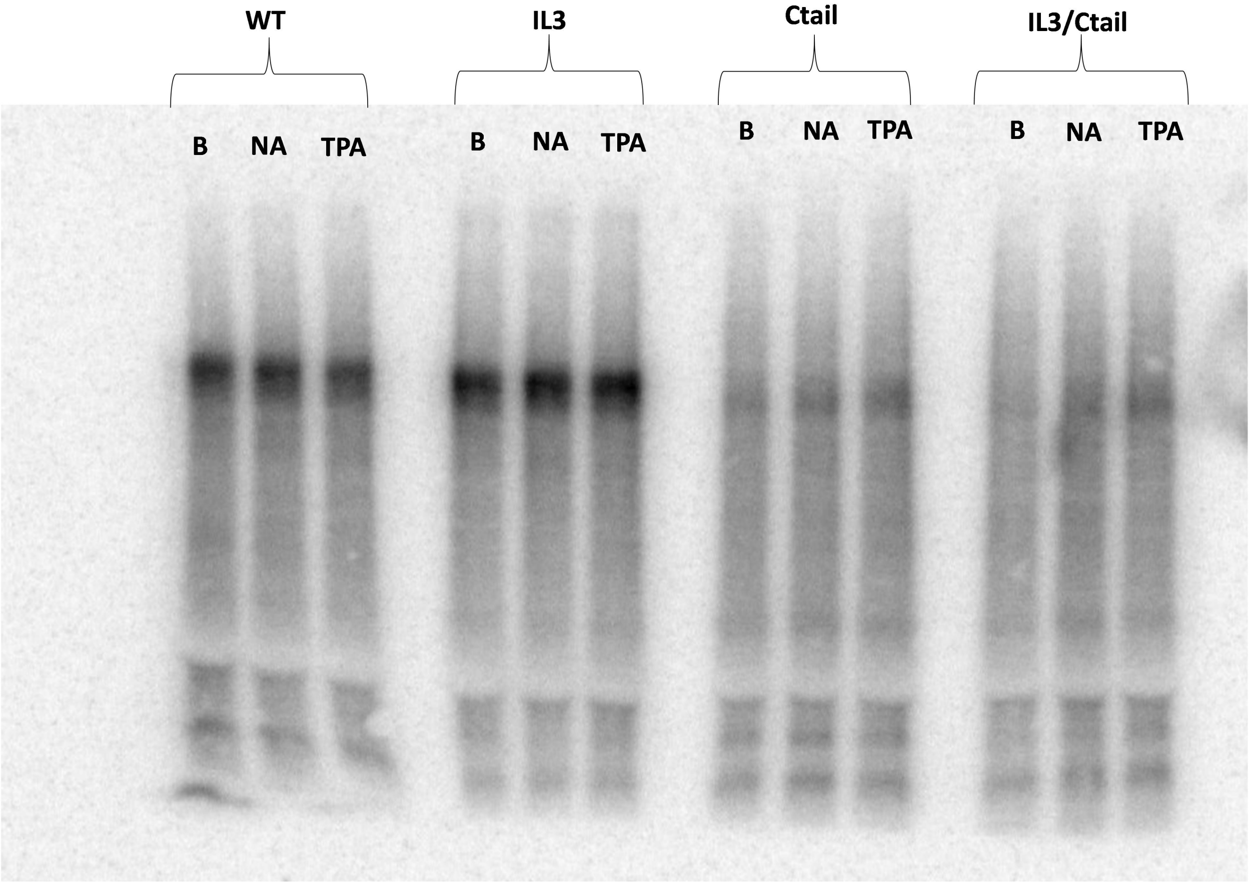

Figure S3. Representative intracellular calcium tracings of cells expressing the WT and mutant α_{1B}-ARs pre-incubated without any agent or for 5 or 10 min with 10 μM NA. Cells were washed and rechallenged with 10 μM NA (homologous desensitization) and subsequently with 1 μM LPA.

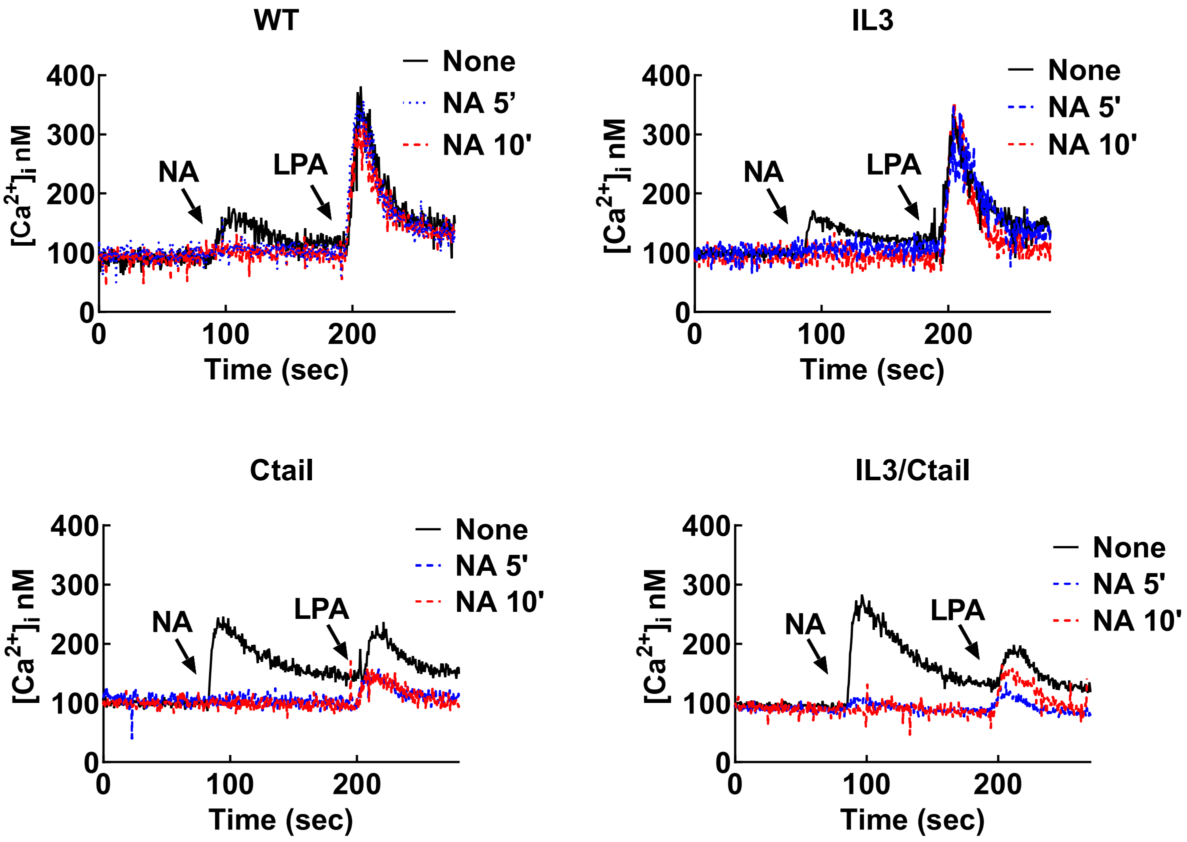

Figure S4. Representative intracellular calcium tracings of cells expressing the WT and mutant α_{1B}-ARs. Cells were pre-incubated without any agent or for 5 or 10 min with 1 μM PMA (heterologous desensitization) and subsequently challenged with 10 μ M NA.

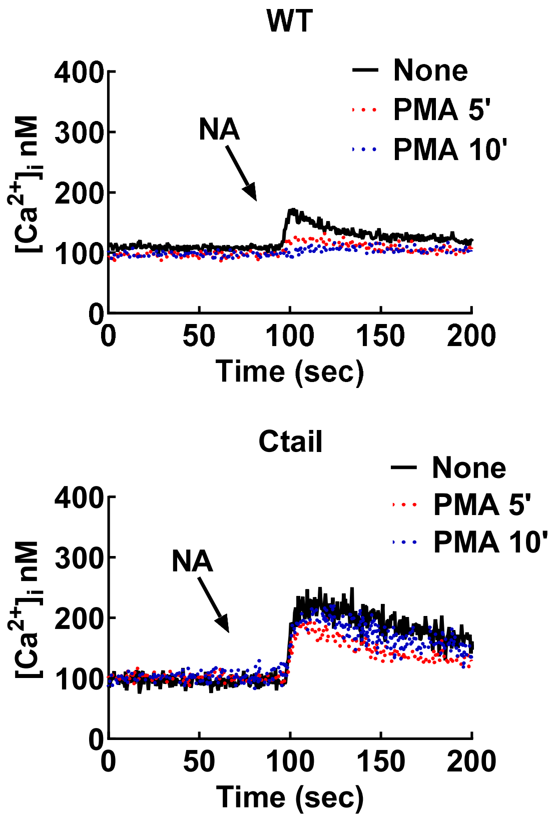

Figure S5. NA-induced ERK phosphorylation (5 and 60 min) in cells expressing the WT α_{1B}-AR, the IL3, the Ctail, or the IL3/ Ctail mutants. The means are plotted, and error bars indicate the S.E.M. of 5 experiments performed on different days using different cell cultures. *** $p < 0.001$ and ** $p<0.05$ vs. cells expressing other receptors, same time of incubation. Representative Western blots are shown above the graph.

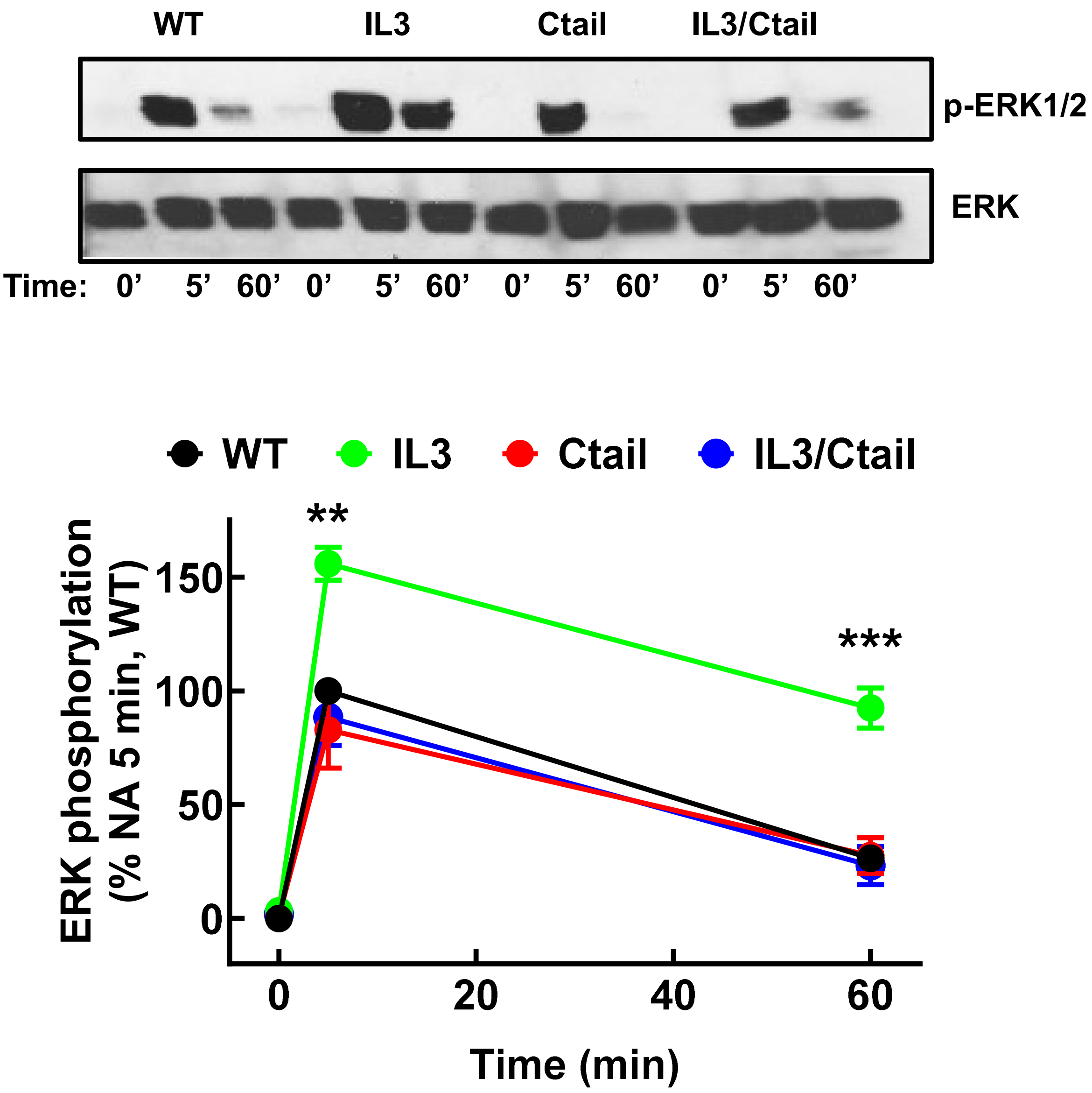

Figure S6. PMA-induced ERK phosphorylation (5 and 60 min) in cells expressing the WT α_{1B}-AR, the IL3, the Ctail, or the IL3/ Ctail mutants. The means are plotted, and error bars indicate the S.E.M. of 5 experiments performed on different days using different cell cultures. *** $p<0.001$ vs. IL3- or Ctail-expressing cells, same time; ** $p< 0.05$ vs. cells expressing other receptor constructs, same time. Representative Western blots are shown above the graph.

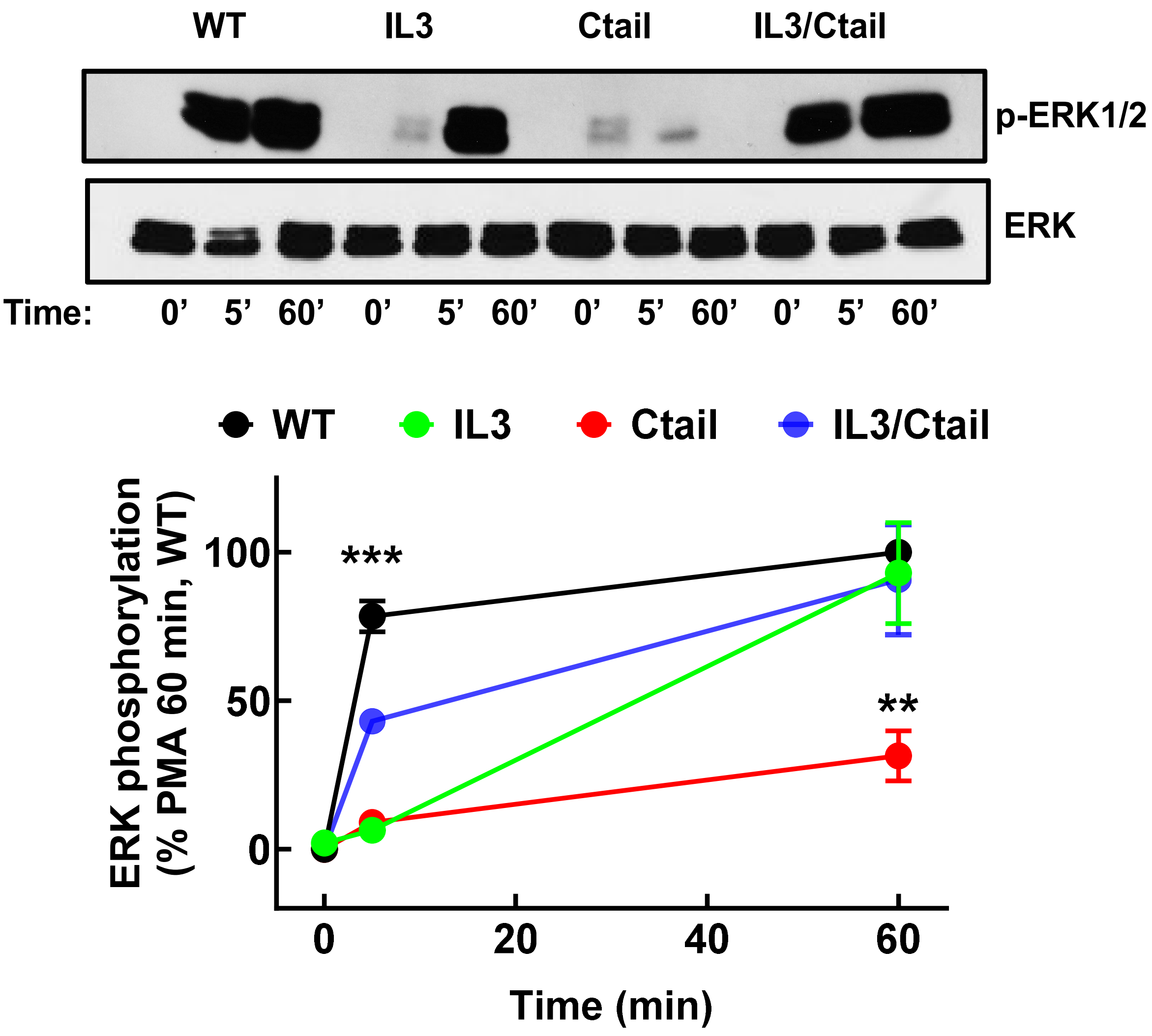

Table S1

Qualitative assessment of the functional repercussion of the distinct α_{1B}-AR mutations on the parameters studied as compared to the wild-type.

Parameter	IL3	Ctail	IL3/ Ctail
Baseline Receptor Phosphorylation	=	↓↓	↓↓/↓↓↓
NA- and PMA-induced Receptor Phosphorylation	=	↓↓	↓↓↓
NA action on $[Ca^{2+}]$ i	EC_{50} ↓<br>Max =	EC_{50} ↓<br>Max =	EC_{50} ↓↓<br>Max ↑↑
[Ca2+] i Desensibilization	NA =<br>PMA ↓	NA =<br>PMA ↓↓↓	NA =<br>PMA ↓↓↓
Receptor internalization	NA ↓<br>PMA ↓	NA ↓↓↓<br>PMA↓↓↓	NA ↓↓↓<br>PMA ↓↓↓
ERK 1/2 phosphorylation	NA ↑↑<br>PMA ↓ delayed	NA =<br>PMA ↓↓	NA =<br>PMA ↓ delayed
